# Lymphoma-Associated Monoclonal Cryoglobulinemic Glomerulonephritis and Relationship with Hepatitis C Virus Infection: A Case Report

**DOI:** 10.1155/2019/7940291

**Published:** 2019-08-18

**Authors:** Sangeeta Mutnuri, Hania Kassem, John Badalamenti, You-Wen Qian, Christopher Marquez, Marjan Afrouzian

**Affiliations:** ^1^Department of Internal Medicine, University of Texas Medical Branch, Galveston, TX, USA; ^2^Department of Pathology, University of Texas Medical Branch, Galveston, TX, USA

## Abstract

We report a case of type I cryoglobulinemic glomerulonephritis in a patient with chronic hepatitis C who presented with acute renal failure. The renal biopsy revealed membranoproliferative GN (MPGN) due to cryoglobulinemia with unexpected monoclonal Kappa restriction on immunofluorescence microscopy, suggesting an underlying hematopoietic malignancy. The bone marrow biopsy revealed presence of marginal zone lymphoma. Our case raises awareness regarding possibility of monoclonality in the renal biopsy of HCV-infected patients and exemplifies the crucial role the renal biopsy plays in detecting lymphoid malignancies where clinical features are ambiguous.

## 1. Introduction

Monoclonal (type I) cryoglobulinemic glomerulonephritis (mCGN) is an uncommon diagnosis, usually reported in association with lymphoproliferative malignancies [1]. Hepatitis C virus (HCV) infection-associated cryoglobulinemia, on the other hand, is a much more common disease, expected to affect the kidney by causing polyclonal (type II) cryoglobulinemic glomerulonephritis (pCGN) [2]. Herein, we introduce an HCV-infected patient who presented with renal failure and in whom the renal biopsy revealed cryoglobulinemic glomerulonephritis (CGN) with monoclonal light chain restriction. The incidences of pCGN and mCGN are compared and the importance of detecting monoclonality in the renal biopsies obtained from HCV-infected patients is entertained.

## 2. Case Report

### 2.1. Clinical History and Initial Laboratory Data

A 47-year-old Caucasian male with a history of HCV infection (genotype 3A) and hypertension was admitted with complaints of shortness of breath, dyspnea on exertion, lower extremity edema, abdominal distention, and decreased urine output for the past two months. Physical examination revealed an elevated jugular venous pulse to 8-10 cm of water, bibasilar crackles, an S3 gallop, bilateral lower extremity edema, and hepatosplenomegaly. He was hypertensive with a blood pressure of 183/111 mmHg. Laboratory tests revealed pancytopenia, hypoalbuminemia, hypocomplementemia, cryoglobulinemia, elevated HCV PCR levels, an elevated creatinine level, and an elevated rheumatoid factor level (Table 1). Urine microscopy revealed dysmorphic red blood cells (RBC) in addition to white blood cell (WBC) and RBC casts. He was also found to have nephrotic range proteinuria (9.5 g in 24 hours).

Retroperitoneal ultrasound only showed a small volume of ascites, 14 cm and 12 cm sized kidneys with no hydronephrosis. HCV-induced cryoglobulinemia was suspected and a kidney biopsy was performed.

### 2.2. Pathology

**Figure 1:**
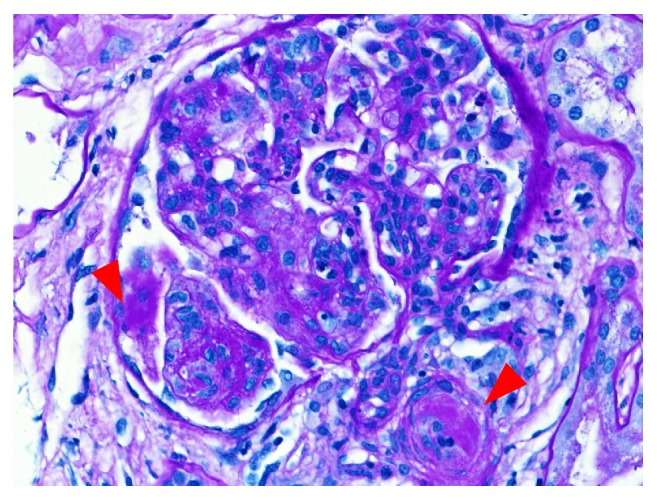
Light microscopy: presence of PAS-positive coagula of cryoglobulin in the glomerular capillaries and arterioles (arrowheads).

Light microscopy (LM): of the twenty-six glomeruli present, one glomerulus showed segmental sclerosis with capsular adhesion. The remaining glomeruli revealed diffuse endocapillary proliferation with lobular simplification and glomerular influx of monocytes, neutrophils, and even eosinophils (one glomerulus). Many glomeruli contained PAS-positive intracapillary coagula of cryoglobulin (Figure 1), and small double contours were observed on silver stain. The interstitium contained sheets of lymphocytes in two foci and a lymphoid follicle with clear germinal center in another focus. Interstitial fibrosis, tubular atrophy, and arterial changes were minimal. One arteriole contained intraluminal cryoglobulin.

**Figure 2:**
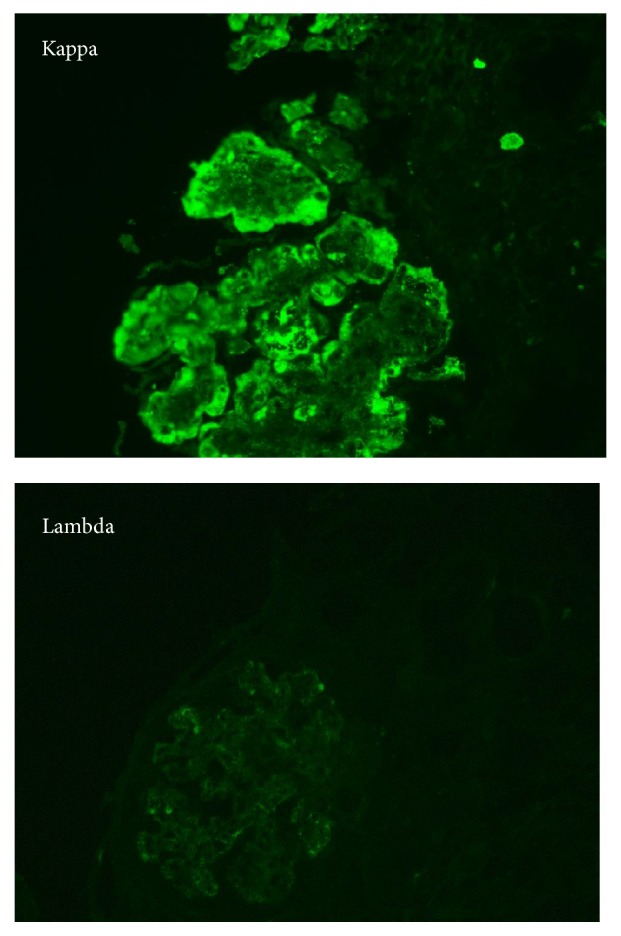
Immunofluorescence microscopy: Kappa light chain deposits within the glomerulus. Lambda light chain is negative.

Immunofluorescence microscopy (IF): eleven glomeruli were present in the specimen processed for IF and revealed the following findings: IgM, Kappa, and C3 (3+) granular staining along peripheral capillary loops (Figure 2); IgG and Lambda (negative to trace) staining in the glomeruli. IgA, C1q, and Albumin were negative. Fibrinogen showed (1+) staining in some glomerular capillaries. Positive controls were satisfactory. As there was more than 2+ difference in intensity between Kappa and Lambda light chains, IgG, IgM, Kappa, and Lambda were repeated and the results of the second run were identical to the first run: presence of IgM with Kappa restriction was confirmed in the deposits.

**Figure 3:**
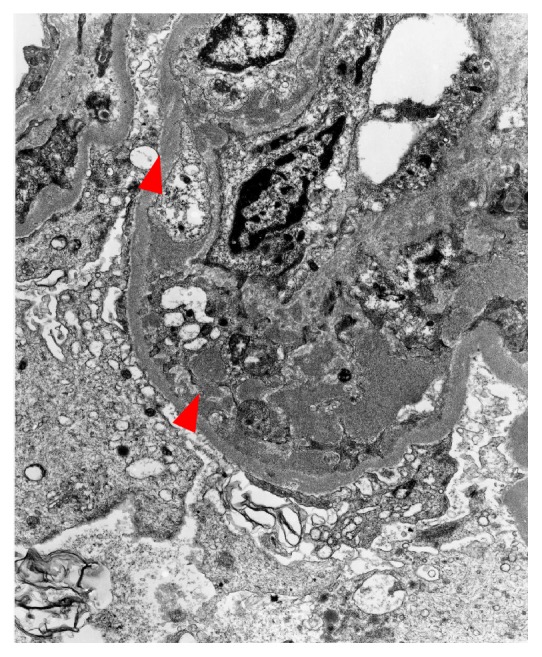
Electron microscopy: subendothelial and subepithelial electron dense deposits of cryoglobulins (arrowheads).

Electron microscopy (EM): endocapillary proliferation, mesangial interposition, and presence of subendothelial and subepithelial electron dense deposits were observed in the single glomerulus present for electron microscopy (Figure 3). On high magnification, characteristic paired and curved organized deposits of cryoglobulin were identified. Occasionally, the deposits revealed a microtubular pattern. Foot processes were focally effaced.

### 2.3. Summary of Findings and Follow-Up

The diagnosis of mCGN (with type III MPGN pattern) was rendered based on the presence of the following findings: features of MPGN (type III), intracapillary coagula of cryoglobulin (by LM), glomerular deposits of IgM with Kappa restriction (by IF), and organized deposits of cryoglobulins (by EM). The pathologist's suggestion regarding the possibility of an underlying B cell malignancy triggered further workup. Serum viscosity was within normal limits, but serum and urine protein electrophoresis revealed IgM monoclonal gammopathy. Computed tomography (CT) detected hepatosplenomegaly associated with retroperitoneal paraaortic lymph node enlargement. Bone survey did not reveal any lytic or blastic lesions; however, bone marrow biopsy results were consistent with bone marrow involvement by low-grade lymphoma, favoring Marginal Zone Lymphoma (MZL). Marrow biopsy showed multiple lymphoid aggregates in the background of trilineage maturing hematopoiesis. The lymphoid aggregates are composed of small-sized lymphoid cells without increased plasma cells or large atypical lymphoid cells. By immunohistochemistry studies, the lymphoid cells in the lymphoid aggregates are mostly PAX-5 and CD20 positive B cells. Plasma cells scattered in the marrow spaces are polyclonal and express both Kappa and Lambda. Flow cytometry detected about 3% of Kappa monoclonal B-cells expressing CD19, CD20, and CD22, but do not express CD5, CD10, or CD103. These findings support a diagnosis of marginal zone lymphoma rather than a lymphoplasmacytic lymphoma or hairy cell leukemia. The patient was initiated on a rituximab/dexamethasone-based regimen, which he tolerated well, with improvement of his renal function, urinary sediment, and proteinuria.

During the course of his hospitalization, hematology and infectious disease were consulted and they favored treating with the above regimen and planned a trial of direct acting antivirals at a later stage. At the most recent follow-up, patient is being continued on the abovementioned therapy and is being monitored by oncology closely.

## 3. Discussion

Our case report represents a combination of rarities and has multiple facets that need to be addressed separately.

### 3.1. Polyclonal and Monoclonal Cryoglobulins

The pathogenesis of HCV infection is closely related to chronic antigenic stimulation. Basically, in response to HCV infection, the virus core particle is captured by the dendritic cells, leading to B cell activation in the marginal zone of the lymphoid follicle, leading to B cell activation. [3] Subsequently, the activated dendritic cells release B lymphocyte activating factor (BAFF), which plays a key role in sustaining the clonal expansion of B cells. In about 20-56% of cases of HCV infection, this process leads to cryoglobulinemia, usually of mixed type (type II cryoglobulinemia) [1]. The mechanism of this polyclonal lymphoid activation is well known: once activated by the HCV antigens, the B cells synthesize large amounts of IgM with rheumatoid factor (RF) activity. These IgM-RF molecules bind HCV viral particles and lead to the formation of immune complexes capable of precipitation when exposed to cold temperatures, called cryoglobulins. These cryoglobulins bind specifically to the endothelial cells via C1q receptors and generate vasoactive peptides from the complement system. This in turn favors recruitment of inflammatory cells and mediates leukocytoclastic vasculitis [3]. While the polyclonal pathway is the most common pathway involved with HCV infection, rarely the infection presents itself with monoclonal cryoglobulinemia. In this case, our patient with chronic HCV antigenemia developed MZL and monoclonal cryoglobulinemia. The cryoglobulins subsequently affected the kidneys causing the uncommon type of MPGN type III.

### 3.2. Cryoglobulinemia and MPGN Type I versus MPGN Type III

About one-third of the patients who have mixed cryoglobulinemia develop renal involvement, which may present itself early on, in the form of an acute nephritic syndrome. More advanced cases typically have nephrotic range proteinuria. The most common renal manifestation and histologic feature of cryoglobulinemia is MPGN, specifically a common subtype of MPGN with subendothelial deposits, known as MPGN type I (not to be confused with type I cryoglobulinemia). Needless to mention that MPGN type I accounts for about 80% of renal biopsies with MPGN [2]. Less often, cryoglobulinemia presents itself in the kidney in the form of MPGN type III, which is the type encountered in our patient's biopsy and is the type that contains both subendothelial and subepithelial deposits. Therefore, our case not only deviates from the usual polyclonal pathway of B cell activation, it also presents itself with the least common histologic patter of MPGN. It is known that immune complex deposits that reach the subepithelial aspect of the glomerulus have less electron negativity (are more cationic) than those that remain in the subendothelial region. Therefore, it is conceivable that at least some of the immune complexes produced by the monoclonal B cell activation in our case have reached the subepithelial region due to their more cationic charge, while others with less cationic charge have been stopped by the electron negativity of the filtration barrier and retained in the subendothelial region. This dual property seen in outpatient can be explained by the viral envelope's dual B cell activation pathways explained above.

### 3.3. The role of Renal Biopsy and The Lesson Learned

Due to complex clinical presentations, there are very few reported cases of mCGN associated with gammopathies and Non-Hodgkin Lymphomas (NHL). [4] In those rare reported cases, it has always been the renal biopsy, specifically the IF findings that have unveiled the ambiguity and dictated the subsequent search for a gammopathy [4]. In a few recent large studies, notably the NCI-SEER, VA health system, and EPILYMPH consortium, a positive, modest increased risk of NHL has been documented in some HCV patients and MZL has been recognized as the most frequent type of lymphoid malignancy encountered [1, 5–7]. This association is further reinforced by several small clinical studies demonstrating regression of lymphomas in response to anti-HCV therapy [8, 9]. Conservative as well as immunosuppressive therapies have been used in a small number of patients with variable outcomes [4]. Multiple therapies have been used for malignancies in the setting of an underlying lymphoproliferative disease including interferon and rituximab therapy. However, mixed results have been obtained on therapies targeting lymphoma. More recently, newer agents, especially direct acting antivirals, have been shown to elicit good lymphoproliferative disease response in HCV associated lymphoproliferative disorders. This trial of interferon-free antiviral therapy is a huge step in establishing a casual relationship between HCV and lymphoproliferative disorders, as well as a guide to assist in treatment decisions [10]. At the present time, treatment decisions are being made based on clinical experience.

## 4. Conclusion

Our case represents a composite of multiple rarities:

1. Monoclonal (type I) cryoglobulinemia which accounts for about 10-15% of total cases of cryoglobulinemias [2].
2. mCGN which is a very rare diagnosis, with fewer than 50 cases reported so far in the literature [11].
3. MPGN type III which is the least common histologic type of MPGN associated with cryoglobulinemia.

While HCV infection and monoclonal B cell proliferation is a notion that is well explored and understood, there are only a few case reports describing the concurrence of mCGN in HCV patients with malignancy [12]. Our case raises awareness mainly among nephropathologists regarding the possibility of monoclonality in the biopsy of HCV-infected patients, especially because polyclonality is the routine finding in these patients. Moreover, it is possible that the actual incidence of mCGN among HCV patients is higher than currently thought as monoclonality can be overlooked in the biopsies: a vigilant diagnosis of mCGN on renal biopsy could be of great assistance in early diagnosis and more effective management approaches, with better patient outcomes. Larger studies will be necessary in order to establish the true prevalence of type I cryoglobulinemic glomerulonephritis in HCV-infected patients.

## Figures and Tables

**Table 1:** Patient's lab results on presentation.

Analyte	Reference Interval	Result
WBC	4.2-10.7 x 10^3^/*µ*L	1.8
RBC	4.26-5.52 x 10^6^/*µ*L	3.1
Hemoglobin	12.2-16.4 g/dL	9.1
Hematocrit	38.4-49.3 %	27.6
Platelets	150-328 x10^3^/*µ*L	86
Blood Urea Nitrogen (BUN)	7-23 mg/dL	42
Creatinine (Cr)	0.60-1.25 mg/dL	2.87
Albumin	3.5-5.0 g/dL	2.8
C3 complement	86-184 mg/dL	55
C4 complement	20.0-59.0 mg/dL	2.8
RF	<20 IU/mL	10700
HCV PCR -1st	IU/ml	890,318 (diluted 1:13)
HCV PCR (1 week later)	IU/ml	2,485,307
HCV PCR (2 weeks later)	IU/ml	6,790,390

Conversion factors for units: BUN in mg/dL to mmol/L, x0.357; Cr in mg/dL to *μ*mol/L, x88.4; C4 complement in mg/dL to *μ*mol/L, x0.05.

HCV PCR result interpretation.

Not detected: target not detected (not the same as negative).

<12 /ml: detected (but not quantifiable).

12-100,000,000 IU/ml.

>100,000,000 IU/ml: >upper limit of quantification.
